# Does Participation in Group Music Activities and Pro-Social Behavior Among College Students Have an Association? A Study of the Interlocking Mediating Effects of Positive Social Connections and Peer Support

**DOI:** 10.3390/bs15010064

**Published:** 2025-01-13

**Authors:** Haohao Yang, Siqin Wang, Xiaolong Chen, Hongfeng Zhang, Cora Un In Wong

**Affiliations:** 1 School of Music, Shaanxi Normal University, Xi’an 710068, China; haohaoyang@snnu.edu.cn; 2 International Design School for Advanced Studies, Hongik University, Seoul 04068, Republic of Korea; wangsiqin@g.hongik.ac.kr; 3 Faculty of Humanities and Social Sciences, Macao Polytechnic University, Macao 999078, China; corawong@mpu.edu.mo

**Keywords:** group music activities, positive social connections, perceived peer support, pro-social behavior, college students

## Abstract

It has been demonstrated that music can be an effective tool for shaping the future of college students. This study aimed to investigate the effects of college students’ participation in group music activities on their pro-social behavior, and for the first time introduced the psychological variables of “positive social connections” and “perceived peer support”. The results of this study show the following: (1) Group music activity participation is positively associated with pro-social behavior. (2) Positive social connections serve as a mediator between college student group music activity participation and college students’ pro-social behavior. (3) Perceived peer support serves as a mediator between college student group music activity participation and college students’ pro-social behavior. (4) Positive social connections and perceived peer support interlock to shape the relationship between group music activity participation and pro-social behavior. This study explores the intrinsic factors of college students’ participation in group music activities to promote their pro-social behavior from the dual perspectives of music psychology and behavioral psychology, provides empirical support for educational practice, emphasizes the importance of group music activities in promoting the development of college students’ pro-social behavior, and provides insights into new perspectives for college students’ overall healthy development.

## 1. Introduction

Young people are the architects of future societies, and their healthy development is directly linked to the sustainable progress of those societies (Hardin & Hackell, 2017). Furthermore, the development of college students’ psychology and behavior is shaped by a variety of complex factors, such as family environment, peer relationships, campus atmosphere, impulsiveness, and moral development, which can affect both positive and negative behaviors (Otto et al., 2021; Ternes et al., 2019; Zhou, 2023). Furthermore, the development of college students’ psychology and behavior is influenced by a multitude of intricate factors, encompassing family environment, peer relationships, campus atmosphere, impulsiveness, and moral laxity (Cutrín et al., 2019; Chen et al., 2024; Pusztai et al., 2017). Scholars, governments, and educators are actively seeking remedies to address the challenges related to the psychological and behavioral development of college students. Traditional and monolithic psychotherapy can no longer adequately address the challenges faced by today’s college students. Consequently, music intervention and music therapy, which blend education with entertainment, have once again become prominent in the realm of education.

As one of the vital means of aesthetic education, music fosters the holistic development of college students by nurturing their aesthetic appreciation, emotional expression, and social skills (Gong, 2023). Music has transcended its role as merely an art form; in modern society, music education is widely recognized as a crucial intervention for regulating college students’ behavioral competencies and elevating their mental well-being. Mature research has demonstrated that engagement in music activities significantly enhances college students’ self-discipline, cooperative spirit, and empathy, thereby mitigating unsocial behaviors (Nwokenna et al., 2023; Hallam, 2010; Clark & Giacomantonio, 2013). Existing research has confirmed the positive influence of music activities on mental health and behavioral norms, yet most studies concentrate on two aspects. Firstly, the substantive impact of music as an intervention tool is explored. For instance, Proter et al. have shown that music can facilitate the regulation of depressive emotions (Porter et al., 2017). Additionally, Archambault et al. propose that “MAP” music therapy effectively regulates emotions among adolescent groups with mental health issues and can intervene in their mental health concerns within inpatient mental health settings (Archambault et al., 2019). Secondly, from psychological and pedagogical perspectives, questionnaires are employed to measure the relationship between music and pro-social behavior. Polzella et al., for example, integrated data from 2008 American families, suggesting that heightened participation in music is conducive to the emergence of pro-social behavior (Polzella & Forbis, 2014).

However, existing research still lacks focus on how music activities influence college students’ pro-social behavior through peer dynamics. Collective music activities, such as choirs, band rehearsals, and concert performances, offer numerous opportunities for interpersonal engagement among college students and facilitate their socialization. By cooperating in these activities, students learn how to coordinate, communicate, and collaborate with others, thereby enhancing their social adaptability (Adderley et al., 2003). More importantly, the voluntary nature of music participation in universities means that most participants actively choose to engage. This implies that most participants share common interests and goals, fostering positive social connections that subtly influence their benevolence towards the world and promote the development of their own pro-social behavior (Davidson & Faulkner, 2010). In group music activities, participants typically collaborate and interact, enhancing emotional resonance and a sense of collective belonging, which in turn strengthens positive social connections. Through joint participation in these activities, college students exhibit more cooperative behaviors within the framework of music, improving their emotional stability and social adaptability. For instance, Draves’s research also indicates that students involved in music learning gain expanded professional knowledge through collaboration and peer guidance, fostering supportive relationships among peers (Draves, 2017). This further supports the link between music activity participation and perceived peer support. Additionally, college students who participate in group music activities often perceive support from their peers, which facilitates emotional expression, cooperation, and conflict resolution, thereby further promoting their pro-social behavior.

Therefore, based on the aforementioned background, this study introduces the variables of positive social connection and perceived peer support, employing multidisciplinary perspectives from behavioral psychology, music psychology, management, and sociology to construct a chained mediation model. The aim is to explore the underlying mechanisms through which participation in group music activities is associated with college students’ pro-social behavior. Through this model, we hope to uncover the potential association between music activities and college students’ behavioral norms, offering substantial theoretical evidence and practical recommendations for higher education institutions, college student development associations, and others. Furthermore, this study seeks to provide a novel perspective for the research on college students’ positive behavioral norms.

## 2. Literature Review and Research Hypothesis

### 2.1. Participation in Group Music Activity and Pro-Social Behavior

Music activity participation refers to the process of individuals or groups interacting through different forms of music activity, covering a wide range of forms from individual activities to collective collaboration. Existing research spans different music forms and participation methods and explores the connotations of this concept from different perspectives. In terms of the chronological context, Stige’s research proposes that music participation in music therapy can be divided into two basic concepts: individual activities and collaborative activities (Stige, 2006). Jo and others believe that participating in musical activities means changing from the role of listener to active participant, actively participating in the perception and production of sound. Jo and others’ research places more emphasis on the initiative of the participants in music (Jo et al., 2013). In short, participation in musical activities is a complex and dynamic process with multiple dimensions. It encompasses individual and collaborative forms of activity and is deeply influenced by the intrinsic motivation and social interaction of the participants.

Group music activities are inseparable from music activities. Simply put, music is a highly group-oriented art form. Apart from some forms such as “solo singing” and “solo performance”, most music activities are group music activities. Group music activities usually include the following five types: first, singing and band performances, including choirs, orchestras, rock bands, and jazz bands, among others (Dunn, 1997); second, concerts and music festivals, including community concerts, school concerts, large-scale music festivals, and outdoor concerts (Snell, 2005); third, improvisation and interactive activities, including improvised concerts and music workshops (Michaelsen, 2014); fourth, karaoke parties and singing competitions (Ruismäki et al., 2013); fifth, traditional and religious music activities, including ethnic music performances and religious music activities (Good et al., 2020). Based on this, this article defines participation in group music activity of the college student group as the process of college students playing, rehearsing, and performing music through group music activities such as choirs, bands, music festivals, and workshops.

The early development and conceptualization of pro-social behavior can be traced back to some basic theories in the fields of psychology and philosophy. Chinese philosopher Confucius and French educator Jean-Jacques Rousseau explored the kindness and social nature of human nature from different perspectives, laying the ideological foundation for later research on pro-social behavior. In 2003, Carlo and Randall and others proposed six dimensions of pro-social behavior (Carlo & Randall, 2002). Their research indicates that pro-social behavior can be divided into six main types, reflecting the motivations and ways in which people exhibit pro-social behavior in different situations. The six dimensions of pro-social behavior are as follows: altruistic behavior, which refers to spontaneous actions intended to help others without expecting anything in return; compliant behavior, which involves responding to explicit requests for help; emotional behavior, which is driven by emotional reactions such as sympathy or empathy, resulting in helping actions; public behavior, which occurs in public settings or is performed to gain public recognition; anonymous behavior, where the helper does not wish to be recognized for their actions; and dire behavior, which is exhibited in emergency or critical situations to provide help. Their view of “six dimensions of pro-social behavior” is recognized by most scholars and widely used to establish research. In 2015, Penner et al. proposed that pro-social behavior can be analyzed and understood at three levels: micro (within the individual), meso (interpersonal relationships), and macro (socio-cultural and organizational environment). The micro level involves the origin of pro-social tendencies and the sources of their changes, focusing on the internal motivation and cognitive processes of individuals. The meso level studies the interactions between helpers and recipients in specific situations, exploring the impact of interpersonal relationships and situations on pro-social behavior. The macro level studies pro-social behavior in groups and large organizations, analyzing the impact of social culture and organizational environments on pro-social behavior. This multi-level framework provides a systematic approach that integrates individual, situational, and socio-cultural factors to understand pro-social behavior and has been widely recognized by scholars. Based on the evolution of the above views, this paper refers to the classic Eisenberg and Miller definition of pro-sociality and defines the pro-social behavior of college students as the behavior that college students voluntarily adopt in different situations to benefit others. This includes actively lending a helping hand when others are in trouble, actively cooperating when someone asks for help, feeling sad about other people’s misfortunes and trying to comfort them, and not hesitating to help in an emergency.

Social Integration Theory emphasizes that individuals can form close social relationships and emotional bonds through interactions and collaborations with others in group activities. This social integration includes the breadth and density of social networks, as well as the emotional support and sense of belonging obtained through these networks. When individuals feel that they are an integral part of the group, they are more inclined to exhibit behaviors that benefit the group, such as helping others and cooperating (Jung et al., 2022). In group music activities such as choirs and bands, college students experience a strong sense of belonging and collective identity through collaboration, joint performances, and mutual support. The process of these activities enhances individual social skills, promotes emotional communication and empathy, and makes college students more likely to understand and care about the emotional states of others, thus exhibiting more pro-social behavior. Group music activities provide positive social support and role models. College students receive support and guidance from peers and mentors in these activities and see and imitate the pro-social behavior of others. At the same time, role sharing and teamwork in music activities cultivate a sense of responsibility and teamwork in college students, making them more willing to contribute to the interests of the group. At the practical level, Clarke and Vuoskoski’s experiment showed that pro-social decisions increased after playing negative videos with emotional music, demonstrating how emotional music enhances empathetic responses, compassion, and pro-social decisions (Clarke et al., 2015). Meanwhile, Polzella and Forbis’s research investigated the relationship between music participation and pro-social behavior, analyzing data from 2008 families. After controlling for the effects of age, race, gender, income, education, marital status, and occupational category, it was found that as concert attendance increased, the likelihood of pro-social behavior was higher, proving the impact of music participation on pro-social behavior (Polzella & Forbis, 2014). Based on this, this paper proposes the following hypothesis:

**H1:**  *Group music activity participation is positively associated with pro-social behavior.*

### 2.2. The Mediating Role of Positive Social Connections and Perceived Peer Support

Social connectedness refers to the process of different individuals establishing connections through different means. For example, Inagaki, T. K. believes that social connectedness is a pleasant, subjective experience of a sense of intimacy and bond with others (Inagaki, 2018). Broadly speaking, social connectedness can be divided into three dimensions: intimate connectedness, relational connectedness, and collective connectedness (Hawkley et al., 2012). Positive social connections refer to the relationships between individuals that are formed through positive interactions and exchanges and are usually reflected in trust, respect, and support for one another.

Existing research shows that synchronous behavior and collective participation in music activities can directly enhance cooperative and sharing behaviors. As Rabinowitch and Meltzoff’s research indicates, shared behaviors in groups of children promote a sense of fairness in resource allocation (Rabinowitch & Meltzoff, 2017). However, this facilitating effect is not limited to direct mechanisms alone, and a growing body of research suggests that social connectedness plays a significant role in the process. Music-making is inherently a highly social experience that can enhance feelings of bonding among participants through emotional synchronization and group interaction. For example, Tzanaki suggests that even simple rhythmic activities performed in synchrony can significantly increase feelings of trust and belonging among participants and that this enhanced social connectedness further creates a positive feedback loop of emotions (Tzanaki, 2022). Positive social connections not only serve as a bridge for music activities to promote pro-social behavior; they can also directly stimulate higher levels of pro-social behavior. Harwood and Wallace’s research found that strong social connections can enhance an individual’s ability to empathize, which in turn promotes an increase in helping behaviors (Harwood & Wallace, 2022). In addition, interventions such as music therapy have been shown to effectively harness this mechanism in at-risk groups, fostering empathy and trust by creating safe social spaces (Rickson & Watkins, 2003). Combining existing research, it can be inferred that group music activities indirectly affect pro-social behavior by strengthening the key variable of positive social connections.

In this study, we focus on college students’ general pro-social behavior tendencies, and in particular on how such tendencies can be cultivated through participation in group music activity. Pro-social behavior in this study is not limited to behavior towards specific group members, but is broadly applicable to all individuals, whether they are peers within the group or strangers outside the group. In this framework, pro-social behavior tendencies are manifested as broader qualities that college students develop through participation in group music activity, which in turn promote their willingness to help or support others in different situations. Specifically, while some studies have emphasized the expression of pro-social behavior within groups (e.g., cooperating to complete tasks and helping teammates overcome difficulties), others have focused on pro-social behavior outside of groups (e.g., lending a helping hand to strangers). This study, however, does not distinguish whether the recipients of pro-social behavior are from the same group. We believe that emotional resonance and empathy in music activities can cross group boundaries and stimulate college students’ tendency to show pro-social behavior to any other person, whether they are familiar peers or strangers. Therefore, the core of this study is to explore this broad-sense pro-social behavior tendency and, in particular, how participation in group music activity can promote college students’ care, support, and help for others. Based on the research of Kok et al., there is a virtuous circle between positive emotions and social connectedness. This emotional connection can effectively promote the expression of pro-social behavior across groups by enhancing individuals’ attention to and empathy for others. Therefore, although this study involved pro-social behavior in different contexts (e.g., toward peers in the same music group and toward strangers outside the group), we did not specifically distinguish between these groups, but instead focused on how college students’ propensity for pro-social behavior in music activities affects their interactions with different others (Kok et al., 2013). Thus, positive social connections, both within and outside groups, serve as a critical bridge between musical activity and pro-social behavior, promoting more positive social interactions and behavior by enhancing emotional energy and social identity.

Interaction Ritual Chain Theory was proposed by sociologist Randall Collins, emphasizing the central role of interactive rituals in shaping human emotions, beliefs, and behaviors in social life. The theory emphasizes that through face-to-face interactions, people can form emotional energy, and this energy can be accumulated and strengthened through continuous interactions (Fine, 2005). The key to the success of an interaction ritual is that participants must be highly attentive and share emotions, creating a sense of empathy and unity, thus forming a strong emotional connection. According to Interaction Ritual Chain Theory, music activities enable people to emotionally resonate and connect through collective participation and synchronized rhythms. This emotional connection enhances the emotional energy of participants and also strengthens their sense of belonging and unity to the group. Positive social connections, by strengthening these emotional bonds, make individuals more willing to adopt pro-social behavior to maintain and strengthen this positive social environment. In other words, when college students feel a strong sense of positive social connection during a musical activity, they are more likely to form a strong emotional resonance. As a result, they are more inclined to exhibit behaviors that help and cooperate with each other in order to continue and consolidate this positive interactive experience (Li et al., 2023). Unlike other studies, this paper introduces positive social connections into the research framework of music and pro-social behavior for the first time. Based on this, this paper proposes the following hypotheses:

**H2:**  *Positive social connections serve as a mediator between college student group music activity participation and college students’ pro-social behavior.*

Peer support is one of the important variables in modern society that explores the psychological aspects of college students. Its concept was first proposed by Lorig and others in 1985. It reflects the interrelationships that exist among individuals in society and is one form of social support (Lorig et al., 1985). Perceived peer support refers to an individual’s subjective understanding and perception of the peer support received. Barrera believes that perceiving social support is the subjective perception of support from others, such as love and understanding, on the individual’s part, that is, the individual’s subjective evaluation of the support received (Barrera, 1986). Perceived peer support is based on this definition and is an individual’s subjective perception and understanding of support from peers. In 1988, Zimet, G. D. and others believed that perceived peer support was an individual’s subjective evaluation and perception of the degree of external support. Since entering modern society, the impact of perceived peer support on mental health has been a research area of great interest (Zimet et al., 1988). Pace and other studies have pointed out that perceived peer support during university years can alleviate internalization problems and mediate the relationship between attachment quality and psychological problems (Pace et al., 2016). College students with secure attachment styles received a higher level of perceived peer support, which significantly reduced the incidence of their internalizing problems. Similarly, Davidson et al. explained the role of perceived peer support from a medical perspective. The research report in the figure pointed out that for adults with severe mental illness, peer support provides an effective form of mental health care, with effects that are comparable to traditional care methods, and even more significant in some respects (Davidson et al., 2005). Wentzel and others explain the role of perceived peer support in social interactions from an educational perspective (Wentzel et al., 2017). He pointed out that in the school environment, students’ perceived peer support is closely related to their motivation and learning efforts. The emotional support provided by peer support can significantly enhance students’ sense of self-efficacy and interest in learning, which is crucial to their academic success. According to a scientific research report by Jacobson et al., in community health services, peer support can significantly enhance the sense of community belonging of service recipients and improve their social interaction skills through relationship building, resource connection, and experience sharing. This form of support is widely used and recognized in mental health and community services (Jacobson et al., 2012).

Interaction Ritual Chain Theory emphasizes that through interactive rituals, individuals form emotional energy and a sense of unity in face-to-face interactions. Perceived peer support plays an important role in group music activities in a number of ways. First, perceived peer support can enhance participants’ emotional energy. This high emotional energy comes from the recognition and encouragement of peers, and it continues to accumulate during interactions, thereby enhancing individuals’ positive emotions. Specifically, when college students feel supported and appreciated by their peers during music activities, they experience higher levels of emotional satisfaction and self-esteem, which further motivates them to actively participate and engage in the activities. Second, peer support provides positive social feedback, strengthening the individual’s status and role identity in the group. According to the theory of an interactive ritual chain, this emotional resonance is the key to the success of interaction, and through the positive feedback mechanism, it further enhances the individual’s sense of belonging and identity. In music activities, peer support and encouragement make participants feel recognized and understood, which resonates emotionally, enhances emotional energy, and at the same time strengthens emotional attachment to the group. Third, individuals who perceive peer support are more likely to follow the group’s behavioral norms and exhibit pro-social behavior. The emotional energy and sense of solidarity formed through interactive rituals are transformed into compliance with group behavioral norms and the expression of positive social behaviors. In other words, perceived peer support motivates individuals to participate more actively in group activities and to be willing to help and cooperate, in order to maintain and strengthen the group’s cohesion and positive interaction atmosphere. Fourth, peer support also further promotes the occurrence of pro-social behavior by enhancing the accumulation of social capital. The theory of an interactive ritual chain suggests that the emotional energy and sense of solidarity accumulated through interactive rituals can be transformed into social capital, which in turn promotes individuals’ pro-social behavior. In group music activities, individuals who perceive peer support are more likely to establish and maintain good social relationship networks. These relationship networks provide individuals with more resources and support, making them more willing to exhibit pro-social behavior. Therefore, perceived peer support plays an important mediating role between participation in group music activities and pro-social behavior by increasing emotional energy, enhancing emotional resonance, promoting compliance with behavioral norms, and accumulating social capital. Unlike other studies, this paper introduces perceived peer support into the research framework of music and pro-social behavior for the first time. Based on this, this paper proposes the following hypothesis:

**H3:**  *Perceived peer support serves as a mediator between college student group music activity participation and college students’ pro-social behavior.*

Group musical activities are typical interactive ritual scenarios in which participants generate emotional resonance through a shared situation (musical performance), and this resonance strengthens positive social connections. The formation of positive social connections is more pronounced when interactions are more frequent and participation is higher. Individuals can more clearly feel the care, collaboration, and support from their peers in interactions with a high degree of emotional synchronization and a common goal (Wiltermuth & Heath, 2009). Peer support also reduces individuals’ feelings of loneliness and insecurity, providing emotional support for pro-social behavior. When individuals perceive peer support, they develop greater confidence in their own social skills. They are more willing to actively give support in return, and in turn become more actively involved in behaviors that benefit the group (Wentzel, 1998).

**Figure 1:**
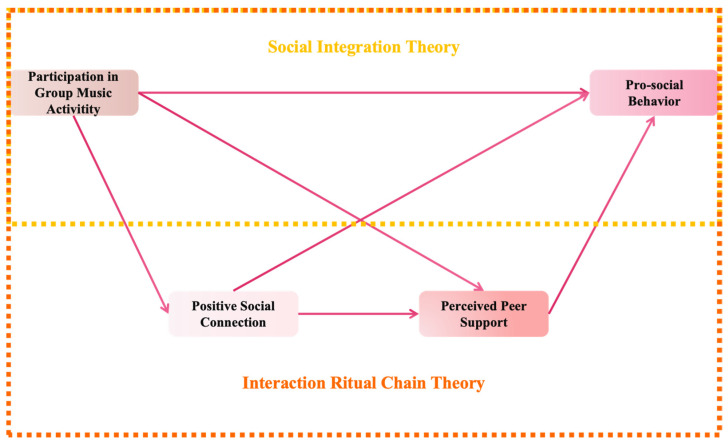
Proposed modeling diagram.

Based on this, this paper proposes a chain mediation model (the proposed model in this paper is shown in Figure 1), that is, college students’ participation in group music activities will have a positive impact on their pro-social behavior through positive social connections and perceived peer support. When college students’ participation in group music activities is higher, the positive impact on positive social connections will be greater, and perceived peer support will also more effectively convey the positive impact of college students’ participation in group music activities on their pro-social behavior. In summary, this paper proposes the following hypothesis:

**H4:**  *Positive social connections and perceived peer support interlock to shape the relationship between group music activity participation and pro-social behavior.*

## 3. Research Design

### 3.1. Sample and Data Collection

This study was conducted through an offline survey, with the data collection period spanning from July to September 2024. The recruiters for the survey included undergraduate students and a small number of first-year graduate students from the participating universities, ensuring that all participants met the criteria of being 18–22 years old and currently enrolled in university. All participants provided informed consent, and the survey process adhered to ethical guidelines, allowing respondents to withdraw from the survey at any time.

The formal survey was conducted at five comprehensive universities across the country, including Shaanxi Normal University, Nanjing University of Science and Technology, and Shanghai Jiao Tong University, among others. These universities were chosen because they are renowned as part of the Chinese “985 Project” and “211 Project” initiatives. Unlike universities with a more localized student body, students at these institutions come from various provinces across China, and the campuses feature a rich variety of group music activities, creating a vibrant musical atmosphere. The survey was conducted at mobile booths set up in areas such as cafeterias and open spaces near music halls, where questionnaires were distributed and data were collected. The questionnaires were randomly distributed across all majors and classes. Prior to the formal survey, a small-scale pilot survey was conducted, gathering 26 valid responses. Focus group discussions were held to identify any unclear or difficult-to-understand questions, and adjustments were made to the initial questionnaire based on issues encountered during the distribution process, as well as the analysis of the 26 pilot responses. This was done to ensure that all items in the final questionnaire were clear and unambiguous. Since the 26 pilot samples were based on the initial version of the questionnaire, they were not included in the final sample.

In this study, a total of 648 questionnaires were distributed, with 578 successfully returned, yielding an initial response rate of 89.2%. To ensure data accuracy and scientific rigor, the returned questionnaires were carefully screened. Questionnaires with duplicate submissions, incomplete answers, response times shorter than 5 min, inconsistencies between answers, or responses with the same option selected for all questions were excluded. After the screening process, 511 valid questionnaires remained, with a final response rate of 78.9%.

The demographics of the respondents are given in Table 1. Among the 511 respondents, 51.9% were female and 48.1% were male. The majority of the respondents were between the ages of 21 and 22 (n = 51.1%). The respondents were predominantly adolescents who have received high-level music education (184, 36.0%), followed by adolescents who have received intermediate-level music education (159, 31.1%). In terms of the number of children in the family, 41.9% of the adolescents came from families with only one child, while 34.1% came from families with two children and 24.1% came from families with three or more children. These data demonstrate that only children make up a larger percentage of the sample. In terms of family geographic location, 41.1% of the adolescents lived in urban areas, 32.1% in suburban areas, and 26.8% lived in rural areas. The distribution shows the majority of adolescents living in urban and suburban areas.

### 3.2. Variable Measurement

All the main variables involved in this paper were measured by drawing on and adopting established and mature scales developed by previous scholars. As the questionnaires in this paper were distributed in Chinese, all the scales used in this study were first translated into Chinese, and then a professional translator, who had obtained a translation certificate, was invited to convert the Chinese and English expressions, and then the team discussed and resolved any discrepancies that arose. The variables were measured on a 5-point Likert-typed scale, where 1 means “strongly disagree or completely disagree” and 5 means “strongly agree or strongly agree”. All questionnaires can be found in Appendix A.

(1) Group Music Activity Participation: In this paper, we used the School Engagement Scale developed by Fredricks et al. to measure students’ behavioral engagement (Fredricks et al., 2004), based on which we revised it according to the Social Activity Engagement for College Students developed by Tang, which contains three dimensions: behavioral engagement, psychological engagement, and social interaction engagement, and to which we added the Group Music Activity Element, which finally resulted in a 4-item questionnaire on group music activity participation (Tang, 2017).

(2) Positive Social Connection: this paper adopted the Self in a Social Context-Social Connectedness Scale (SSC-SC) developed by (Lok & Dunn, 2022), on the basis of which appropriate changes were made according to the characteristics of the participants in this paper, and four items were retained as the measurement question items in this paper.

(3) Perceived peer support: The Perceived Social Support Scale (PSSS) developed by (Zimet et al., 1988) and later revised by Chinese scholars was used, and it consists of three dimensions: family support, peer support, and other support. In this study, the four items of the questionnaire about peer support were selected as the question items for measuring the perception of peer support.

(4) Pro-social behavior: Using the Pro-social Tendencies Measure (PTM) scale developed by (Carlo & Randall, 2002) in their study ’Development of a Pro-social Behavior Measure for Late Adolescents’, this research examined the correlates and constructs of pro-social behavior in late adolescents. The PTM assesses six types of pro-social behavior: altruistic, compliant, emotional, dire, public, and anonymous. However, considering the cultural context of this study, particularly the restrictive nature of the research scenario in Chinese music culture, the dimensions of ’dire’ and ’public’ were excluded through focus group discussions. Furthermore, a four-item scale covering the dimensions of altruistic, compliant, emotional, and anonymous behaviors was evaluated and modified based on the integration of previous research.

## 4. Data Analysis and Results

This study applied SPSS 28.0 and AMOS 24.0 for multi-stage data analysis, including descriptive data analysis, confirmatory factor analysis (CFA), structural equation modeling (SEM), and mediation effect analysis. The reliability and validity of the research model were first assessed using CFA, and then the hypotheses were tested using SEM and mediation effect analysis.

### 4.1. Measurement Model

The reliability and validity of the data in a test model can be assessed based on several statistical indicators, including Cronbach’s Alpha, Composite Reliability (CR), and Average Variance Extracted (AVE). Cronbach’s Alpha thresholds at values of 0.7 or higher are generally acceptable, indicating good internal consistency (Fornell & Larcker, 1981). In the data results for Table 2, the Alpha values for all constructs (PGMA = 0.904, PSC = 0.874, PPS = 0.914, PB = 0.795) were well above this threshold, indicating that the items in each construct reliably measured the same underlying concepts.

CR values greater than 0.7 are generally considered acceptable, with higher values indicating better reliability (Hair et al., 2020). The constructs of the data showed CR values (PGMA = 0.905, PSC = 0.877, PPS = 0.921, PB = 0.800) that exceeded this threshold, reinforcing the assessment of reliability provided by Cronbach’s Alpha and confirming the consistency and reliability of the constructs.

The AVE measures the average proportion of variance in the observed variables that can be attributed to their underlying factors, thus assessing the convergent validity of the constructs (Farrell, 2010). The AVE values for all constructs (PGMA = 0.705, PSC = 0.641, PPS = 0.744, PB = 0.501) met or exceeded the criterion of 0.5, which implies that the constructs explained more than half of the variance of their indicators. It indicates that a significant proportion of the observed variables is due to the hypothesized underlying constructs. Meanwhile, factor loadings also indicate the correlation between the observed variables and their underlying constructs. High loadings on the data results indicate that these terms strongly indicate constructs.

Therefore, the results in Table 2 indicate that the constructs and their corresponding items are reliable and valid for measuring participation in group music activities, positive social connections, perceived peer support, and pro-social behavior.

Discriminant validity is based on the AVE values for each dimension. As shown in Table 3, the squared correlations between the constructs and the AVE for each construct on the diagonal are represented. Discriminant validity is supported when the AVE for each construct is greater than the squared correlation between that variable and any other construct. The reality of Table 3, where the discriminant validity of constructs is confirmed because the variance between each construct and its metric is greater than the variance with other constructs, is critical to ensuring the completeness and applicability of the constructs in the research framework.

Since the data were collected through a questionnaire, we conducted Harman’s single-factor test to check for possible common method bias. The results showed that all extracted factors explained 73.751% of the total variance, which represents that the extracted factors captured a large portion of the variance, a positive sign (Fuller et al., 2016). The first factor explained 40.018% of the first variance, indicating that common methodological bias was not a significant issue in our study.

### 4.2. Structural Model

Structural modeling includes the evaluation of path coefficients and model fitness. We bootstrapped 2000 times to explore the significance of each path. As shown in Table 4, the overall fit measure of the model was acceptable (Bentler, 1990). Values of CMIN/DF less than 3 generally indicate a good fit, and values closer to 1 are desirable, suggesting that the model does not deviate significantly from the observed data. Values of GFI greater than 0.95 are generally considered indicative of a good fit, suggesting that it captures the variance and covariance in the data well. Values of AGFI greater than 0.95 generally indicate a good fit, suggesting that it captures the variance and covariance in the data well. Given the complexity of the model, AGFI values above 0.9 are usually considered good values. RFI also above 0.95 indicates that the model is an improvement over the baseline model adjusted for degrees of freedom. Values of IFI, TLI, and CFI close to 1 indicate a perfect fit, which suggests that the model fits the data well. Values of RMSEA less than 0.05 are usually considered to indicate that the model is an excellent approximation of the observed data without overfitting.

The results for the data in Table 4 show excellent fit on all metrics, indicating that the model is well specified and represents the underlying data structure well. This robust model fit supports the validity of the conclusions drawn from the model parameters and the overall theoretical framework.

**Figure 2:**
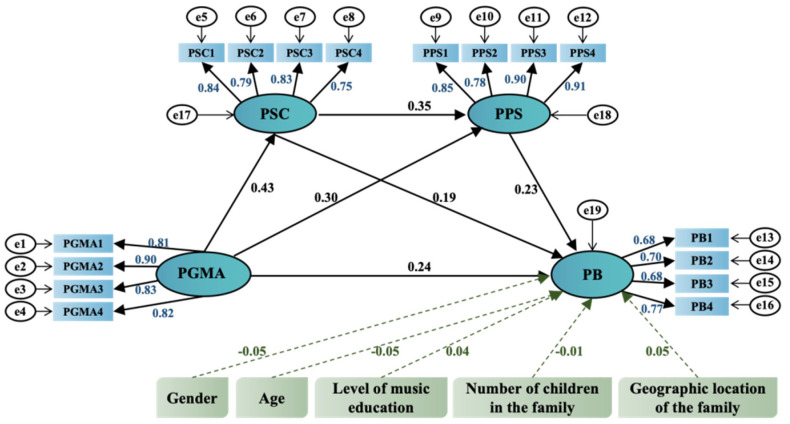
SEM testing results.

The standardized path coefficients and significance levels for each path were examined (Wright, 1960), as shown in Figure 2 and Table 5. This study first tested the effect of PGMA on the other three variables. The results showed that PGMA was significantly correlated with PSC (β = 0.430, *p* < 0.001), PGMA was positively correlated with PPS (β = 0.297, *p* < 0.001), and PGMA was positively correlated with PB (β = 0.244, *p* < 0.001). As expected, PSCs were significantly associated with PPS (β = 0.349, *p* < 0.001); PSCs were positively associated with PB (β = 0.244, *p* < 0.001) and pro-social behavior (β = 0.192, *p* < 0.001). Meanwhile, the coefficient of the linear term between PPS and PB was positive and statistically significant (β = 0.228, *p* < 0.001). The data supported all paths in the model, indicating a strong and statistically significant relationship between the constructs. Given the high C.R. values and very low *p*-values, these findings are robust, suggesting empirical solid support for the hypothesized model.

In addition to these primary constructs, the model incorporated five control variables—gender, age, level of music education, number of children in the family, and geographic location of the family—to examine their effects on PB. The results indicated that none of the control variables had a statistically significant impact on PB. Specifically, gender (β = −0.047, *p* = 0.284), age (β = −0.047, *p* = 0.285), level of music education (β = 0.037, *p* = 0.399), number of children in the family (β = −0.014, *p* = 0.752), and geographic location of the family (β = 0.050, *p* = 0.254) did not reach significance, suggesting that these demographic and contextual factors were not influential in predicting PB in this model.

### 4.3. Chain Mediation Effect in the Research Model

The mediating effects analysis in the research model confirmed all four hypotheses about the effects of participation in group music activities on adolescents’ pro-social behavior, with positive social connections and perceived peer support as mediating factors (Hayes, 2017). The results of the chain mediation effect are presented in Table 6.

The direct effect of PGMA on PB (Estimate = 0.244, *p* < 0.001) was statistically significant and positive, consistent with our hypothesis that PGMA enhances college students’ pro-social behavior, and thus, H1 is supported. The indirect effect (Estimate = 0.083, *p* = 0.002) through PSC was significant, indicating that PSC mediated the relationship between PGMA and PB. This supports the idea that positive social connections mediate the relationship between participation in group music activities and college students’ pro-social behavior. Thus, H2 is supported. The indirect effect only through PPS (Estimate = 0.068, *p* < 0.001) indicated that PPS was a significant mediator. This finding suggests that participation in group music activities, as influenced by PGMA, positively affects college students’ pro-social behavior. Hence, H3 is supported. The chain mediation effect (Estimate = 0.034, *p* < 0.001) through PSC and PPS was statistically significant. This result highlights a continuous mediation effect whereby PGMA increases PSC, which in turn increases PPS, collectively leading to an increase in PB; hence, H4 holds.

The total effects, integrating direct and mediated pathways, further bolster these findings, showing substantial overall impacts of PGMA on PB through these mediating variables. Each pathway, whether direct or mediated, contributes to explaining the dynamic relationships proposed in the hypothesis, emphasizing the importance of positive social connections and perceived peer support for participation in group music activities elicited by adolescents for aspects of pro-social behavior.

## 5. Discussion

The main findings of this study suggest a positive relationship between group music activities and pro-social behavior among university students. This relationship is mediated by positive social connections and perceived peer support. These results are consistent with our hypothesis and align with existing research, further deepening our understanding of the social and psychological effects of group music activities.

This study confirms the impact of group music activities on pro-social behavior among university students, resonating with the findings of Rickson and Watkins, who explored the role of music therapy in promoting pro-social behavior in adolescents (Rickson & Watkins, 2003). However, unlike studies that focus on specific forms of music therapy interventions, this research highlights the potential value of music activities as a daily social medium, emphasizing its broad applicability and naturalness. Additionally, since group music activities typically involve repeated rehearsals, the overall time commitment is longer compared to other group-based activities (Brendell, 1996). Extended exposure to group music activities fosters greater rapport, inclusivity, and harmony among participants. It also helps transform peers who initially engage in music activities together into genuine friends over time (Pennill & Timmers, 2022; Ginsborg & King, 2012; Miell & Littleton, 2008). This undoubtedly further reinforces the positive role of group music activities in shaping peer relationships among college students.

Moreover, this study reveals the important role of positive social connections and perceived peer support in the relationship between group music activities and pro-social behavior through a chain mediation model. This finding expands the application of Social Support Theory and further supports Langeland and Wahl’s argument regarding the crucial role of social support in mental health and behavioral development (Langeland & Wahl, 2009). This study specifically highlights that group music activities, through their non-competitive and collaborative interaction environment, enhance the emotional bonds, trust, and cooperation skills among university students. The improvement of these positive social traits significantly promotes the expression of pro-social behavior.

In addition, the findings of this study complement those of other empirical research. For example, Staiano et al. demonstrated that adolescents who participated in group dance games experienced significant improvements in subjective health and peer support. This study further suggests that music activities can directly influence pro-social behavior by enhancing adolescents’ perceptions of positive social connections and peer support (Staiano et al., 2018). Similarly, Burland emphasized the importance of ensemble participation in promoting group belonging, well-being, and identity formation. This study, in turn, demonstrates how these psychological dimensions directly impact pro-social behavior during the college student phase (Burland, 2021).

In conclusion, this study not only validates the positive impact of group music activities on college students’ pro-social behavior but also elucidates the underlying mechanisms through a chain mediation model. By integrating Social Support Theory, this study highlights the potential value of group music activities in enhancing students’ mental health and social functioning. These findings offer theoretical support and practical insights for the fields of education and psychological intervention.

## 6. Conclusions and Implications

### 6.1. Conclusions

This study combines music psychology with behavioral psychology to explore the impact of college students’ participation in group music activities on their pro-social behavior. For the first time, it introduces two psychological variables—“positive social connection” and “perceived peer support”—and uses the peer perspective to understand pro-social behavioral tendencies. By constructing a chain mediation structural equation model, this study analyzes the mediating mechanisms between students’ participation in group music activities and their pro-social behavior. The results indicate the following: (1) Group music activity participation is positively associated with pro-social behavior. (2) Positive social connections serve as a mediator between college student group music activity participation and college students’ pro-social behavior. (3) Perceived peer support serves as a mediator between college student group music activity participation and college students’ pro-social behavior. (4) Positive social connections and perceived peer support interlock to shape the relationship between group music activity participation and pro-social behavior.

### 6.2. Implications

Based on the findings of this study, group music activities demonstrate a positive impact on promoting pro-social behavior among college students, with this process being mediated by positive social connections and perceived peer support. Therefore, it is recommended that universities actively promote the popularity and participation of group music activities in curriculum design and extracurricular activity arrangements. By organizing diverse group music activities, students’ emotional bonds and cooperation abilities can be enhanced, fostering mutual trust among students and subsequently facilitating the development of their pro-social behavior.

Furthermore, this study reveals the profound impact of the interactive environment in group music activities—especially the non-competitive and collaborative atmosphere—on students’ mental health and behavioral development. Therefore, educators and mental health professionals should consider group music activities as an effective psychological intervention tool, playing an active role in improving college students’ perception of social support and enhancing their mental health. Universities can establish long-term, stable platforms for music activities, such as regular music rehearsals or social music events, to provide more opportunities for students to support and help each other in collective activities, thereby achieving the dual goals of improving mental health and behavioral outcomes.

Finally, this study, in conjunction with social support theory, further validates the role of group music activities in promoting mental health. To maximize the social–psychological effects of group music activities, universities should focus on the long-term and regular nature of activity design. By implementing appropriate organizational structures and management mechanisms, each participant can find a sense of belonging and support within the group. This also contributes to improving the overall psychological quality of college students and provides valuable insights for diverse strategies in mental health education and student development within universities.

## 7. Limitations and Future Directions

Although this study provides some understanding of the relationship between college students’ participation in group music activities and pro-social behavior, there are still some limitations. First, due to the constraints of offline research sample collection, this study mainly focuses on universities in certain cities in China. As a result, the research sample may not fully represent the entire population of college students in China. Additionally, the sample does not include college students from different ethnicities and backgrounds in other countries, which limits the representativeness and generalizability of the findings. Second, this study uses cross-sectional data, which prevents longitudinal tracking of college students’ psychological states. Third, the chain mediation model used in this study provides only one perspective on pro-social behavior. Therefore, other factors influencing college students’ pro-social behavior have not been explored. Future research should focus on global college student samples from different countries and use longitudinal research methods to better understand the psychological development of college students. Future studies should also introduce variables such as “musical self-expression” and “musical empathy” to more comprehensively examine the psychology of college students in music activities. These issues will be further explored in future research.

## Figures and Tables

**Table 1:** Demographics of respondents (n = 511).

Measure	Items	Frequency	Percentage
Gender	Male	246	48.1%
	Female	265	51.9%
Age	21–22	261	51.1%
	20	123	24.1%
	19	66	12.9%
	18	61	11.9%
Level of music education	Have received high-level music education (have received music competition certificates)	184	36.0%
	Have received intermediate-level music education (e.g., professional piano lessons, etc.)	159	31.1%
	Received basic music education (school music lessons, etc.)	112	21.9%
	Have not received specialized music education	56	11.0%
Number of children in the family	Three children or more	123	24.1%
	Two children	174	34.1%
	Only child	214	41.9%
Geographic location of the family	Urban	210	41.1%
	Suburban	164	32.1%
	Rural	137	26.8%

**Table 2:** Confirmatory factor analysis.

Construct	Items	Mean	SD	Standard Loadings	Alpha	CR	AVE
Participation in group music activities (PGMA)	PGMA1	3.409	1.278	0.809	0.904	0.905	0.705
	PGMA2	3.540	1.266	0.899			
	PGMA3	3.554	1.227	0.830			
	PGMA4	3.474	1.217	0.817			
Positive social connections (PSCs)	PSC1	3.808	0.997	0.837	0.874	0.877	0.641
	PSC2	3.816	0.955	0.788			
	PSC3	3.847	1.000	0.828			
	PSC4	3.877	1.121	0.747			
Perceived peer support (PPS)	PPS1	3.685	1.190	0.853	0.914	0.921	0.744
	PPS2	3.646	1.100	0.783			
	PPS3	3.843	0.889	0.902			
	PPS4	3.659	1.154	0.907			
Pro-social behavior (PB)	PB1	3.413	1.321	0.677	0.795	0.800	0.501
	PB2	3.597	1.162	0.702			
	PB3	3.609	1.410	0.676			
	PB4	3.665	1.288	0.771			

**Table 3:** Test results of discriminant validity.

Construct	PGMA	PSC	PPS	PB
PGMA	**0.840**			
PSC	0.388 **	**0.801**		
PPS	0.408 **	0.427 **	**0.863**	
PB	0.378 **	0.353 **	0.372 **	**0.708**

Note: Bolded diagonal numbers are AVE square roots, others are correlation coefficients, ** indicates *p* < 0.01.

**Table 4:** Model fitting index.

CMIN/DF	GFI	AGFI	NFI	RFI	IFI	TLI	CFI	RMSEA
1.451	0.967	0.954	0.971	0.965	0.991	0.989	0.991	0.03

**Table 5:** Structural equation modeling results of the hypotheses.

Path			STD. Estimate	S.E.	C.R.	*p*-Values
PSC	←	PGMA	0.430 ***	0.040	8.776	***
PPS	←	PGMA	0.297 ***	0.048	6.117	***
PPS	←	PSC	0.349 ***	0.060	7.018	***
PB	←	PGMA	0.244 ***	0.049	4.309	***
PB	←	PSC	0.192 ***	0.062	3.313	***
PB	←	PPS	0.228 ***	0.050	3.976	***
PB	←	Gender	−0.047	0.037	−1.072	0.284
PB	←	Age	−0.047	0.078	−1.07	0.285
PB	←	Level of music education	0.037	0.039	0.844	0.399
PB	←	Number of children in the family	−0.014	0.05	−0.316	0.752
PB	←	Geographic location of the family	0.05	0.048	1.142	0.254

Note: ***: *p* < 0.001.

**Table 6:** Test for chain mediating effects.

Parameter		Estimate	Lower	Upper	*p*-Values
Direct effect	PGMA-PB	0.244 ***	0.121	0.365	***
Indirect effect	PGMA-PSC-PB	0.083 ***	0.034	0.141	*
	PGMA-PPS-PB	0.068 ***	0.031	0.115	***
	PGMA-PSC-PPS-PB	0.034 ***	0.017	0.057	***
Total effect	PGMA-PSC-PB	0.327 ***	0.211	0.437	***
	PGMA-PPS-PB	0.312 ***	0.192	0.423	***
	PGMA-PSC-PPS-PB	0.278 ***	0.161	0.393	***

Note: ***: *p* < 0.001; *: *p* < 0.05.

## Data Availability

The original contributions presented in this study are included in the article; further inquiries can be directed to the corresponding author.

## Appendix A. Questionnaire

Questionnaire								
Personal Basic Information	Iterms		Options					
	Gender		1	Male			**◎**	
			2	Female			**◎**	
	Age		1	21–22			**◎**	
			2	20			**◎**	
			3	19			**◎**	
			4	18			**◎**	
	Level of music education		1	Have not received specialized music education			**◎**	
			2	Received basic music education (school music lessons, etc.)			**◎**	
			3	Have received intermediate level music education (e.g., professional piano lessons, etc.)			**◎**	
			4	Have received high-level music education (have received music competition certificates)			**◎**	
	Number of children in the family		1	Three children or more			**◎**	
			2	Two children			**◎**	
			3	Olny child			**◎**	
	Geographic location of the family		1	Urban			**◎**	
			2	Suburban			**◎**	
			3	Rural			**◎**	
Category	Measurement items			1	2	3	4	5
Participation in group music activities (PGMA)	PGMA1	I actively participate in group music activities organized by the school.		**◎**	**◎**	**◎**	**◎**	**◎**
	PGMA2	I will practice and perform carefully in group music activities.		**◎**	**◎**	**◎**	**◎**	**◎**
	PGMA3	I will work with my classmates to ensure that the music activities go smoothly.		**◎**	**◎**	**◎**	**◎**	**◎**
	PGMA4	I feel that participating in group music activities enhances my musical skills and social skills.		**◎**	**◎**	**◎**	**◎**	**◎**
Positive social connections (PSC)	PSC1	I feel that I am part of a community of friends.		**◎**	**◎**	**◎**	**◎**	**◎**
	PSC2	I find connections with friends enjoyable and fulfilling.		**◎**	**◎**	**◎**	**◎**	**◎**
	PSC3	I feel accepted and understood when I interact with others.		**◎**	**◎**	**◎**	**◎**	**◎**
	PSC4	My friends make me feel valued and respected.		**◎**	**◎**	**◎**	**◎**	**◎**
Perceived peer support (PES)	PES1	My friends support me when I am in trouble.		**◎**	**◎**	**◎**	**◎**	**◎**
	PES2	I feel I have friends I can count on to share my worries and troubles.		**◎**	**◎**	**◎**	**◎**	**◎**
	PES3	I have friends who will offer help and advice when I need it.		**◎**	**◎**	**◎**	**◎**	**◎**
	PES4	I have friends who care about my feelings and needs.		**◎**	**◎**	**◎**	**◎**	**◎**
pro-social behavior (PB)	PB1	I will do my best to help those in trouble, even if I don’t know them.		**◎**	**◎**	**◎**	**◎**	**◎**
	PB2	I will try to cooperate if someone asks for my help.		**◎**	**◎**	**◎**	**◎**	**◎**
	PB3	I will feel sorry for someone’s misfortune and try to comfort them.		**◎**	**◎**	**◎**	**◎**	**◎**
	PB4	In an emergency, I will not hesitate to help.		**◎**	**◎**	**◎**	**◎**	**◎**
(1: Strongly disagree, 2: Disagree, 3: Neither agree nor Disagree, 4: Agree and 5: Strongly agree)								
